# Characterization of cell wall components of wheat bran following hydrothermal pretreatment and fractionation

**DOI:** 10.1186/s13068-015-0207-1

**Published:** 2015-02-15

**Authors:** Zara Merali, Samuel R A Collins, Adam Elliston, David R Wilson, Andres Käsper, Keith W Waldron

**Affiliations:** The Biorefinery Centre, Institute of Food Research, Norwich Research Park, Colney, Norwich, NR4 7UA UK; Biogold OÜ, Lossi 19A, 12616 Tallinn, Estonia

**Keywords:** Arabinoxylans, Phenolics, Hydrothermal pretreatment, Simultaneous saccharification and fermentation, Wheat bran

## Abstract

**Background:**

Pretreatments are a prerequisite for enzymatic hydrolysis of biomass and production of ethanol. They are considered to open up the plant cell wall structure by altering, moving or solubilizing lignin and hydrolyzing a proportion of hemicellulosic moieties. However, there is little information concerning pretreatment-induced changes on wheat bran cell wall polymers and indeed on changes in cell wall phenolic esters in bran or other lignocellulosic biomass. Here, we evaluate polymeric changes (chemical and physical) as a result of selected hydrothermal pretreatment conditions on destarched wheat bran using controlled polymer extraction methods. Quantification of cell wall components together with soluble oligosaccharides, the insoluble residues and ease of extractability and fractionation of biomass residues were conducted.

**Results:**

Pretreatment solubilized selected arabinoxylans and associated cross-linking ferulic and diferulic acids with a concomitant increase in lignin and cellulosic glucose. The remaining insoluble arabinoxylans were more readily extractable in alkali and showed considerable depolymerization. The degree of arabinose substitution was less in xylans released by higher concentrations of alkali. The recalcitrant biomass which remained after pretreatment and alkali extraction contained mostly cellulosic glucose and Klason lignin. Pretreatment generated small but insignificant amounts of yeast-inhibiting compounds such as furfural and hydroxymethyl furfural.

As such, simultaneous saccharification and fermentation of the hydrothermally pretreated bran resulted in increased ethanol yields compared to that of the control (97.5% compared to 63% theoretical).

**Conclusion:**

Hydrothermal pretreatment of destarched wheat bran resulted in degradation and depolymerization of the hemicellulosic arabinoxylans together with some breakdown of cellulosic glucose. This was accompanied by a significant reduction in the cross-linking phenolic acids such as ferulic and diferulic acids. The results suggest that hydrothermal pretreatment enhances enzymatic digestibility of the cellulose not only by depolymerization and solubilization of the hemicelluloses but by breakdown of interpolymeric phenolic cross-links between the remaining insoluble polymers. This allows easier access of hydrolytic enzymes by opening or loosening of the cell wall thus resulting in enhanced saccharification of cellulose and subsequent fermentation to ethanol. The reduction in cinnamic acids by selected breeding or biotechnological approaches could provide a useful basis for improved saccharification and fractionation of wheat bran polysaccharides.

## Background

Wheat bran consists of the outer coat (pericarp, testa and aleuron layers) of the wheat grain (*Triticum aestivum* L). Wheat bran is separated from the other parts of the wheat kernel by milling, and the chemical composition of wheat bran predominantly comprises non-starch polysaccharides (approximately 38%), starch (approximately 19%), protein (approximately 18%) and lignin (approximately 6%), with the non-starch polysaccharides being approximately 70% arabinoxylans, approximately 19% cellulose and approximately 6% β-(1,3)/β-(1,4)-glucan [1]. It also contains significant amounts of phenolic acids such as ferulic and *p*-coumaric acids [2] which are esterified to arabinofuranosyl residues [3]. The structural and physical properties of these arabinoxylans are largely determined by the degree, type and distribution pattern of the substitutions along the xylan backbone [4]. Furthermore, the substitution pattern and the interpolymeric cross-linkages such as those mediated through phenolics acids will affect their digestibility [5] and enzymatic degradation [6]. Wheat bran is an important by-product of the cereal industry produced in vast quantities worldwide, and it is estimated that more than a 100 million tons of bran are available in the European Union [7]. Global demand for sustainable resources means that there is an increasing socioeconomic pressure to ensure complete utilization of such feedstocks [8]. Industrial wheat bran represents 14%–19% of the wheat grain and has the potential to serve as a low-cost feedstock for the production of renewable energy or chemicals. It is envisaged that utilization of both the starch and hemicellulosic/cellulosic part of wheat bran would greatly facilitate potential applications in a biorefinery concept. However, hydrolysis of bran using hemicellulolytic and cellulolytic enzymes is not adequate to liberate sufficient simple sugars; therefore, a range of physical or chemical pretreatments are required [9].

Compared with other pretreatments, the advantages of hydrothermal pretreatment (HT) include a significantly lower environmental impact, lower capital investment, the avoidance of chemicals and low by-product generation [9]. It is now clear that hot water and related thermophysical pretreatments enhance saccharification of lignocellulose by disrupting the cell wall matrix [10-12], thereby improving the accessibility of cellulases to the cellulosic microfibrils.

Recent research has indicated that the pretreatment-induced changes in the cell walls are highly complex and differ from species to species and tissue to tissue reflecting the variation in wall chemistries [13,14]. More detailed information on chemical changes during pretreatment and enzymolysis has resulted from the use of antibodies to cell-wall polymer epitopes in wheat straw [15] and poplar [16] providing further information on the protective roles of different polymers throughout the process. It is becoming evident that the mechanisms of pretreatment are far from being fully resolved and differ between different lignocellulosic sources.

Most research on the impacts of pretreatment on cell walls involves studies on plant stem and leaf tissues [13-16]. There is relatively little information available on how pretreatments affect the polymeric nature of cell walls of wheat bran which surrounds the seed and has much less of a structural role in the plant [17]. This is of significance if the non-cellulosic polysaccharides are to be additionally exploited e.g. in food production or materials. Alkaline extraction/fractionation of wheat bran arabinoxylans have been reported using hydrogen peroxide [18,19] and potassium hydroxide [20,21]. Some enzymatic hydrolysis of arabinoxylan fractions from wheat bran have also been described [22]. However, although pretreatment of wheat bran for bioethanol production has been studied (e.g. [23]) and pretreatment conditions have been used for extraction of arabinoxylans of varying quality [24], there is limited knowledge of pretreatment-induced changes on wheat bran cell walls or alterations at a polymeric level relevant to fractionation. Since arabinoxylans in wheat bran are physically and chemically associated with each other and with other cell wall components such as lignin, an alteration in any of these components is likely to affect the accessibility of cellulases during saccharification as shown in cell walls of other biomass sources [25]. Previously, we reported the effects of hydrothermal pretreatment on the degradation and sequential alkali extractability of wheat straw cell wall polymers [17]. We demonstrated that under conditions that augment enzymatic saccharification, significant changes occur in the extractability/fractionation and chemistry of cell wall hemicelluloses, lignin and cross-linking phenolics. Of particular note was the reduction in the molecular weight (Mw) of the remaining arabinoxylans and a considerable reduction in ferulic and diferulic acids. In the present paper, the polymeric changes (chemical and physical) are reported as a result of hydrothermal pretreatment (180°C and 200°C) on wheat bran. Using controlled polymer-extraction/fractionation methods [26], we have evaluated pretreatment-induced changes in the constituent polymers and associated phenolic cross-linkages. In order to account for the fate of cellulose and hemicellulose through the solid and liquid streams, mass balance calculations were conducted together with the quantification of yeast-inhibiting compounds such as furfural and hydroxymethylfurfural (HMF). The impact of the pretreatments on the production of ethanol from bran was evaluated by simultaneous saccharification and fermentation (SSF) process using a commercial enzyme (CTec2®) and Ethanol Red® yeast.

## Results and discussion

### Physical effect of hydrothermal pretreatment on wheat bran

**Figure 1:**
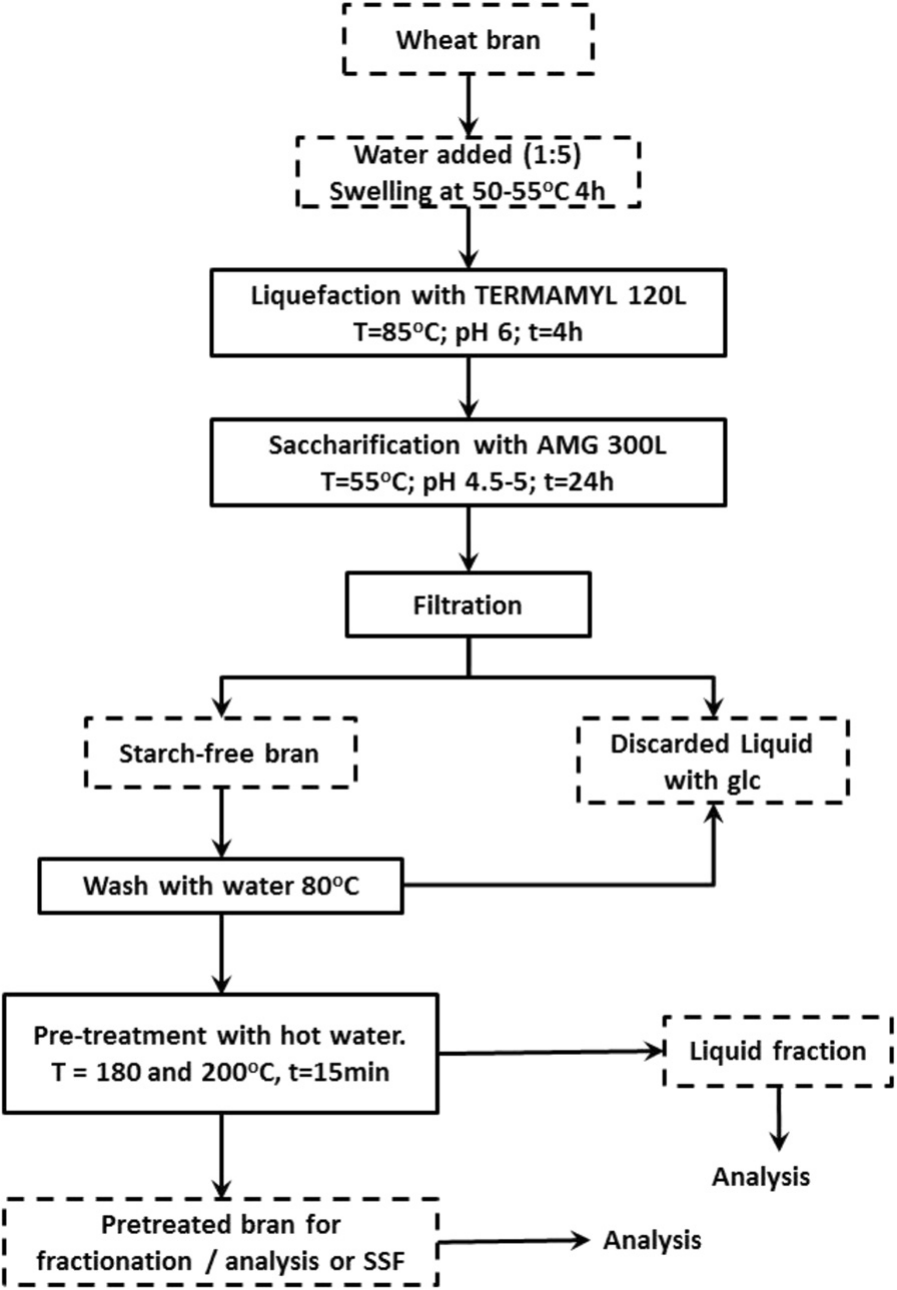
**Pretreatment and destarching procedure developed for wheat bran.**

**Figure 2:**
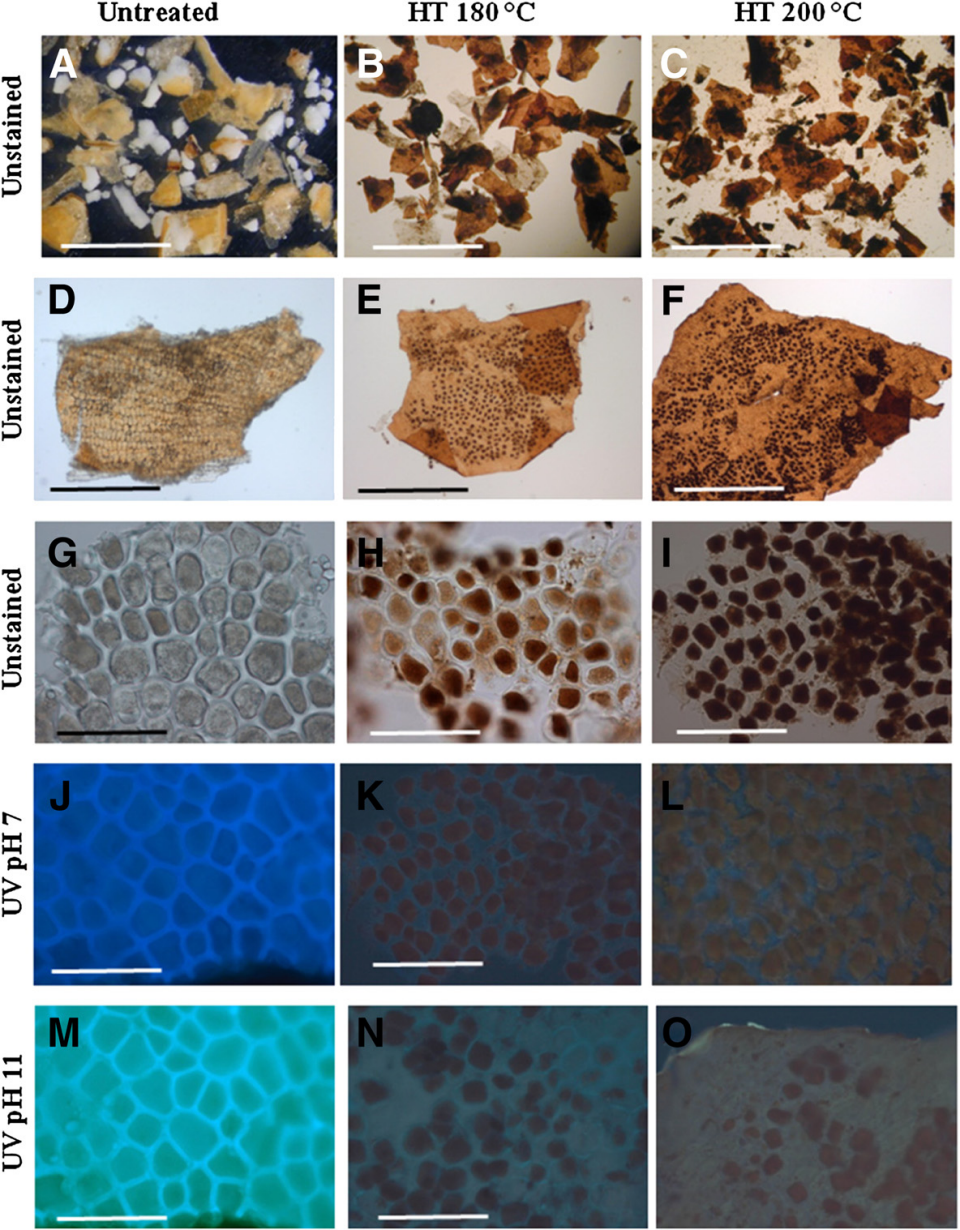
**Photomicrographs of control and pretreated wheat bran.** Images of unstained bran **(A-C)** and at higher magnification **(D-I)** showing colouration due to Maillard reactions in the pretreated materials. Under UV, lignified native bran tissues autofluoresce blue at neutral pH **(J)** whilst at alkaline pH, cinnamic acid and related moieties autofluoresce green **(M)**. However in the HT samples, this autofluorescence is visibly reduced. Bars: 1 mm **(A-C)**; 0.5 mm **(D-L)**; 100 μm **(G-O)**.

Wheat bran was destarched and pretreated as shown in Figure 1 after which it was extracted as an alcohol insoluble residue (AIR). Low magnification imaging of the unstained AIRs exhibited layers of wheat bran such as the aleurone (the outer layer of the endosperm) and the outer pericarp, together with the starchy endosperm (Figure 2A-C). Microscopy depicted the aleurone as the most abundant layer present in wheat bran and in agreement with previous work on separated bran layers which showed that the largest fraction consisted of the aleurone (62%) followed by the pericarp layer (27.1%) [2]. The most noticeable visual effect of hydrothermal pretreatment (HT) on the aleurone layer compared to the untreated control (Figure 2D) was the brown coloration (Figure 2E,F) which intensified with the severity of treatment. A closer examination depicted the presence of lipids which (colourless in the untreated aleurone layer; Figure 2G) had darkened probably as a result of oxidation occurring during the pretreatment (Figure 2H,I). Under UV irradiation, lignin-rich material has been shown to autofluoresce blue at neutral pH and the presence of phenolics (such as ferulic acid) is indicated by turquoise/green autofluorescence at pH 11 [5]. A strong positive result was observed for the untreated, control aleurone (Figure 2J,M) indicating the presence of phenolics and related moieties esterified to these cell walls. However, the HT aleurone layers showed no such autofluorescence suggesting a degradation and reduction in the levels of fluorescing moieties (Figure 2K,L,N,O).

### Effect of hydrothermal pretreatment on cell wall composition of wheat bran

**Figure 3:**
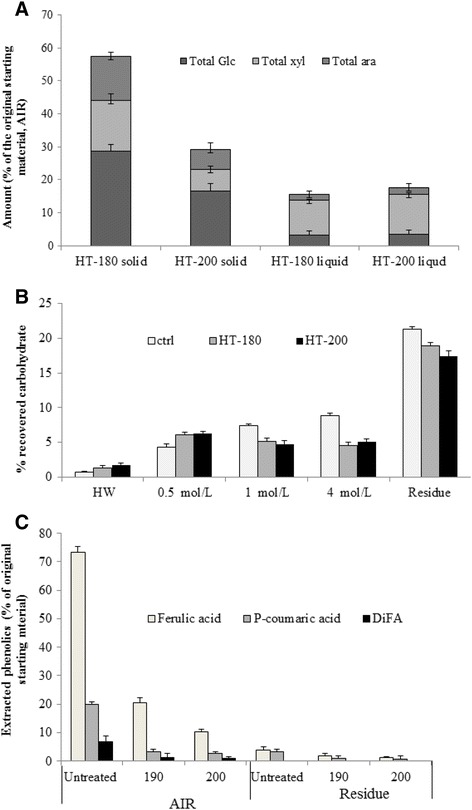
**Cell wall composition of wheat straw (A) showing the total amount of key carbohydrates recovered in the solid biomass and pretreatment liquors as a function of original starting material and (B) total recovery of carbohydrates present in alkali fractions and (C) selected phenolic acids from the control and pretreated wheat bran.** *PT* pretreated, *pCA p*-coumaric acid, *FA* ferulic acid, *DiFA* diferulic acids.

Table 1 shows the effect of hydothermal pretreatment (HT) on the gross compositions of alcohol insoluble residues (AIRs) with detailed sugar mass balance and compositions shown in Table 2 and Figure 3A. Untreated wheat bran AIR consisted of 77% carbohydrate. Most of this carbohydrate comprised of glucose, xylose and arabinose, reflecting 18.5% (cellulosic) glucan and 51.8% (xylan) hemicellulose and is in agreement with previous reports [18,20]. A large proportion of the insoluble hemicelluloses were solubilized as a result of HT (40% and 51% at 180°C and 200°C, respectively; Table 2) and a smaller but not inconsequential amount of (cellulosic) glucose (Figure 3A). This is caused by acid hydrolysis consistent with the high temperature and mildly acidic conditions as observed previously for instance in wheat straw [27,17] and arabinoxylan extraction from destarched wheat bran [24].

**Table 1:** **Compositional data of the (destarched) control and hydrothermally pretreated (HT) wheat bran (analysed in alcohol insoluble residue (AIR))**

**Component**	**Content (% w/w original starting material)**		
	**Untreated**	**HT-180°C**	**HT-200°C**
DM	89.0 ± 0.4	19.1 ± 0.7	18.3 ± 1.7
Cellulose	18.5 ± 2.1	21.9 ± 2.0	24.2 ± 2.6
Hemicellulose (total):	51.8 ± 4.2	31.2 ± 1.7	26.0 ± 2.1
Starch	4.1 ± 0.5	1.9 ± 0.1	0.8 ± 0.0
Phenolic acids (esters)	1.5 ± 1.2	0.6 ± 0.3	0.4 ± 0.1
Klason lignin	10.8 ± 1.3	12.4 ± 2.4	14.7 ± 1.5

**Table 2:** **Carbohydrate composition (μg/mg dry weight) of untreated control and hydrothermally pretreated wheat bran following sequential extraction**

	^**a**^ **Yield (g)**	**Rhamnose**	**Fucose**	**Arabinose**	**Xylose**	**Mannose**	**Galactose**	**Glucose**	**GlcA**	**Total**
Ctrl AIR	2.02	1.4 ± 0.3	0.7 ± 0.1	228 ± 3	291 ± 3	5.5 ± 0.1	15.2 ± 1.2	185 ± 2	42.8 ± 0.5	769 ± 3
Ctrl HW	0.02	nd	nd	139 ± 2	228 ± 3	8.3 ± 0.3	17.6 ± 1.1	140 ± 2	35.1 ± 0.4	568 ± 4
Ctrl 0.5 mol/L	0.15	nd	nd	167 ± 3	223 ± 4	1.7 ± 0.1	8.1 ± 0.3	162 ± 1	32.3 ± 0.3	594 ± 5
Ctrl 1 mol/L	0.18	nd	nd	190 ± 4	433 ± 6	2.9 ± 0.2	8.3 ± 0.4	177 ± 2	31.1 ± 0.3	841 ± 8
Ctrl 4 mol/L	0.20	nd	nd	196 ± 4	466 ± 5	1.1 ± 0.1	10.4 ± 1.6	173 ± 2	25.2 ± 0.2	871 ± 6
Ctrl residue	1.00	nd	nd	83 ± 2	103 ± 3	0.8 ± 0.1	3.3 ± 0.8	204 ± 2	30.6 ± 0.5	425 ± 6
180°C AIR	2.05	nd	nd	126 ± 2	188 ± 2	5.5 ± 0.6	14.1 ± 1.6	220 ± 4	40.6 ± 1.1	593 ± 7
180°C HW	0.05	nd	nd	119 ± 2	192 ± 3	6.1 ± 0.8	15.3 ± 1.2	185 ± 3	32.1 ± 0.5	550 ± 6
180°C 0.5 mol/L	0.16	nd	nd	228 ± 3	290 ± 2	1.0 ± 0.2	6.4 ± 1.1	196 ± 2	27.8 ± 0.3	749 ± 6
180°C 1 mol/L	0.17	nd	nd	190 ± 2	289 ± 3	1.8 ± 0.3	6.7 ± 1.1	85 ± 1	29.2 ± 0.3	601 ± 5
180°C 4 mol/L	0.24	nd	nd	75 ± 1	185 ± 2	1.2 ± 0.4	9.3 ± 2.0	86 ± 1	21.6 ± 0.4	379 ± 3
180°C residue	0.76	nd	nd	63 ± 1	97 ± 1	0.7 ± 0.1	2.6 ± 0.5	265 ± 2	28.3 ± 0.3	457 ± 5
200°C AIR	1.98	nd	nd	101 ± 1	152 ± 2	5.5 ± 0.5	13.5 ± 1.1	242 ± 3	37.8 ± 0.8	551 ± 6
200°C HW	0.07	nd	nd	92 ± 2	107 ± 2	5.9 ± 0.5	13.8 ± 1.2	184 ± 1	24.2 ± 0.5	427 ± 4
200°C 0.5 mol/L	0.19	nd	nd	207 ± 4	218 ± 3	1.4 ± 0.4	5.8 ± 0.3	153 ± 1	27.9 ± 0.7	613 ± 6
200°C 1 mol/L	0.19	nd	nd	102 ± 2	238 ± 4	2.2 ± 0.2	6.8 ± 0.2	75 ± 1	29.7 ± 0.6	453 ± 4
200°C 4 mol/L	0.25	nd	nd	76 ± 1	161 ± 3	1.3 ± 0.1	8.2 ± 0.7	88 ± 2	28.9 ± 0.8	364 ± 3
200°C residue	0.71	nd	nd	59 ± 1	95 ± 2	0.4 ± 0.1	1.6 ± 0.2	291 ± 3	28.0 ± 0.5	476 ± 5

Residue represents the insoluble material after fractionation. Values are expressed as means of duplicate analysis ± SD.

*GlcA* glucuronic acid, *Ctrl* untreated control, *nd* none detected.

^a^Yield is the dry weight of fraction recovered after sequential extraction of the AIR in hot water (HW) and progressively stronger alkali (0.5, 1 and 4 mol/L KOH).

The untreated bran AIR contained 10.8% lignin (w/w) in agreement with previously reported data of 10% [28]. The pretreatment-induced reduction in insoluble carbohydrate resulted in a concomitant increase in the level of Klason lignin to nearly 15% (w/w; Table 1) at 200°C. Ferulic and *p*-coumaric acid were the most abundant phenolic acids present in wheat bran (details in Table 3). The amount of phenolic acids recovered in the untreated control was ca 1.5%. These were reduced during hydrothermal pretreatment, consistent with previous studies for instance in steam exploded barley bran where the effect of high temperatures (above 220°C) resulted in decreasing levels of ferulic acid compared to *p*-coumaric acid [29]. However, a significant proportion (0.6% and 0.4% at 180°C and 200°C, respectively) remained esterified to the arabinoxylans. A considerable amount of ferulic acid was present in the dimer form (19% of the total; Table 3), indicating a high degree of cell wall cross-linking [30]. The presence of ferulic acid dehydrodimers is likely to have a profound effect on the functional characteristics of the cell walls as indicated by previous studies on wheat bran [2] and Chinese water chestnut [5,31]. The reduction in interpolymeric cross-linking such as those mediated via diferulic acid would be expected to affect the ease of extraction of arabinoxylans and enhance the accessibility of cell wall degrading cellulases during saccharification. Interestingly, whilst the pretreatment-related reduction in the measured levels of ferulic and diferulic acid corresponded to the decrease in alkaline autofluorescence under UV (Figure 2M-O), this was not the case for lignin. Figure 2J-L shows a reduction in autofluorescence of lignin whilst the measured Klason lignin increased (Table 1). The loss of fluorescence may have resulted from the degradation of fluorescing moieties during pretreatment. It may also be the case that other wall components such as carbohydrate and protein breakdown products have interacted with the lignin resulting in chemical change or perhaps quenching autofluorescence. Indeed, previous studies have indicated that in some biomass, pretreatment results in the formation of “pseudo lignin” [32,33] which would be quantified by the Klason methodology. Such uncharacterized material may not exhibit fluorescence associated with native lignin. In addition, much lignin is known to be solubilized under pretreatment conditions [25] and it is possible that such solubilization involves the loss or relocation of autofluorescing components.

**Table 3:** **Phenolic acid composition (μg/mg) of wheat bran before (in AIRs) and after alkaline sequential extraction**

	***p*** **-coumaric acid**	**Ferulic acid**	**8,5′-DiFA**	**5,5′-DiFA**	**8-** ***O*** **-4′-DiFA**
Ctrl AIR	2.6 ± 0.5	9.7 ± 0.4	0.9 ± 0.1	0.9 ± 0.2	1.0 ± 0.4
190°C AIR	1.0 ± 0.2	3.6 ± 0.4	0.3 ± 0.1	0.5 ± 0.1	0.7 ± 0.0
200°C AIR	0.8 ± 0.2	2.7 ± 0.3	0.2 ± 0.4	0.2 ± 0.0	0.4 ± 0.0
Ctrl residue	0.4 ± 0.1	0.5 ± 0.1	nd	nd	nd
190°C residue	0.2 ± 0.0	0.5 ± 0.1	nd	nd	nd
200°C residue	0.1 ± 0.0	0.2 ± 0.0	nd	nd	nd

Values are expressed as means of duplicate analysis ± SD.

*nd* none detected.

### Characterization of AIRs, sequentially extracted fractions and the insoluble residues

Sequential extraction in progressively strong alkali provides a basis for comparing changes in cell walls during physiological development as well as physicochemical/thermal processing [34]. AIRs were sequentially extracted in hot water followed by progressively stronger alkali (in degassed aqueous solution and in the presence of NaBH_4_ to minimize alkaline peeling) which progressively extracts the non-cellulosic cell wall polysaccharides. Table 2 (and Figure 3B) shows the recoveries and yields of total carbohydrate whereas the total phenolic compositions are shown in Table 3 (and Figure 3C). Sequential extraction of the control AIR solubilized 49% of the material (Table 2). In contrast, alkaline extraction of the HT AIR samples resulted in the solubilization of larger quantities of material (63% and 64% at 180°C and 200°C, respectively; Table 2).

Most solubilization of AIR carbohydrate was effected by 1 and 4 mol/L alkali treatments, particularly in the 200°C HT material. This resulted in relatively less material remaining in the insoluble residues from HT biomass (Figure 3B). The main carbohydrate released from the AIR during alkali fractionation comprised arabinoxylan hemicelluloses (Table 2). The extracted polymer also retained phenolic acid groups which had resisted HT and saponification (Table 3, Figure 3C). Very little soluble material was recovered in the hot water (HW) fraction of the non-pretreated wheat bran, and what was solubilized consisted mostly of arabinose; xylose and non-cellulosic glucose (Table 2). Yields of approximately 9% have been obtained for water-extractable material from purified wheat bran using more aggressive treatment at 100°C, 30 min [18]. Nevertheless, the results indicate that most wheat bran arabinoxylans are not extractable in water probably because of their structural features and/or association with each other and other cell wall components. After pretreatments, slightly larger quantities of hot water soluble material was released from the AIRs (<4%; in the 200°C HT). The relative degree of xylan branching could be inferred from the ratio of arabinose to xylose and in the HT polymers this decreased (0.78 in untreated AIR compared to 0.66 in 200°C AIR, respectively), consistent with a decrease in water solubility. The level of arabinose substitution further decreased with increasing concentrations of KOH to 0.42 in untreated and 0.38 in 200°C HT in 4 mol/L extracts. This reflects a higher degree of xylan-cellulose hydrogen bonding in the latter polymers, thereby requiring more concentrated alkali for their solubilization. The decrease in water solubility is consistent with previous studies on wheat bran [35] and brewers’ spent grain [20]. The isolated fractions contained uronic acid units probably derived from both glucuronoxylan and pectic polymers (indicated by the presence of small quantities of rhamnose) which are thought to have originated from epidermal cells of the inner pericarp [2].

The insoluble residues consisted mainly of arabinoxylan and cellulosic material (Table 2). A significant level of xylose remained in the cellulose-rich residue after the 4 mol/L KOH extraction (approximately 20% of the total carbohydrate, Table 2), indicating that some xylan was cross-linked to other alkali-stable wall components. These residues were also analysed for esterified cinnamic acid derivatives (data in Table 3). The bulk of the phenolics, especially ferulic acid, was lost as a result of the pretreatment (Figure 3C) and further due to sequential alkaline extraction which hydrolyzes the labile ester bond between the phenolics and arabinose. However, a significant amount (approximately 7% of the total in untreated, Table 3) remained esterified to the isolated arabinoxylan and recalcitrant residue. The most prominent phenolics were ferulic acid, followed by *p*-coumaric acid and dehydrodimers of ferulic acid. In the HT AIRs, the reduction in phenolics correlated with increasing severity of temperature; and in terms of the remaining phenolics present in the HT residues, only ferulic acid (thought to be involved in cell-to-cell adhesion) and *p*-coumaric acid (which is thought to be ether linked with the matrix polymers and/or with lignin) were detected.

### Molecular weight (Mw) distribution of solubilized xylan following fractionation

**Figure 4:**
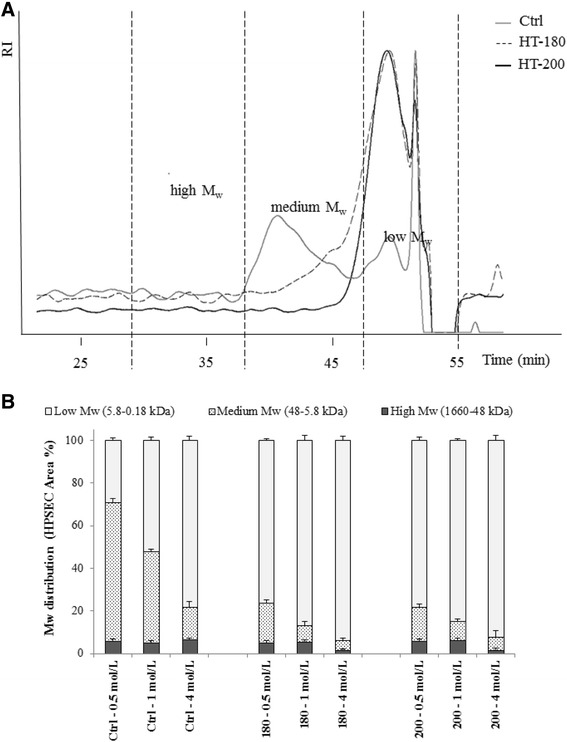
**Molecular weight (M**_**w**_ **) distribution of saccharides present in extracted fractions of the untreated control (ctrl), and HT treated at 180°C and 200°C wheat bran (A) depicting representative spectra obtained following HPSEC and (B) HPSEC area percentages based on peak areas calculated using dextran standards.**

Molecular masses of xylans solubilized during fractionation and following hydrothermal pretreatment were evaluated by high-performance size exclusion chromatography (HPSEC) in combination with RI detection. The representative spectra are presented in Figure 4A. Three dominant peaks were identified using elution profiles of standard controls (Figure 4A,B); high Mw range (1,660 to 48 kDa), medium (Mw range 48 to 5.8 kDa) and low (Mw range 5.8 to 0.18 kDa). The smaller but nonetheless significant peaks (approximately 6% of the total area) observed at high Mw range (1,660 to 48 kDa) may have originated from the adherent wheat endosperm (Mw 850 kDa of the alkaline extract) [36]. Generally, the relative amount of medium Mw xylans decreased in stronger alkali with a concomitant increase in the amount of low Mw xylans (Figure 4B). Thus, the xylan moieties remaining in the bran cell walls after hydrothermal pretreatments are of a lower molecular weight indicating that during milder conditions larger structures of xylan are solubilized and at extreme conditions these high Mw xylans are subsequently hydrolyzed to medium and low Mw xylans. This is in agreement with the xylan solubility of various feedstocks producing polymers of varying Mw sizes for instance during hot water pretreatment of wheat straw [27,17], eucalyptus wood [37] and corn cobs [22]. Furthermore, in all bran preparations (including the control), the xylans extracted by the higher alkali concentrations exhibit lower molecular weights than those extracted in less concentrated alkali. The ease of alkali extraction is likely to reflect the degree of xylan substitution, whether it be other sugars (e.g. arabinose) or acetyl groups. The latter have been highlighted during microarray polymer profiling of pretreated wheat straw polysaccharides [15].

There is varied information available on the Mw profiling of alkali-extracted wheat bran hemicelluloses [18,19,21]. Maes and Delcour [18] also observed two peaks at high and low Mw sizes, and the xylan components are of the order reported here for wheat bran. However, in contrast, Hollmann and Lindhauer [19] and Zhang et al. [21] only observed one peak at a Mw range of 100–110 kDa corresponding to high Mw range (116–48 kDa) in this study. Both studies used relatively aggressive extraction methods; peroxidation, which is highly degradative, and 24% KOH followed by HPSEC in DMSO could result in more destructive solubilization and include aggregates derived from lignin. In the present study, alkaline degradation was minimized by utilizing degassed water and the addition of 20 mmol/L NaBH_4_ prior to extraction.

### Evaluation of pretreatment liquor

**Figure 5:**
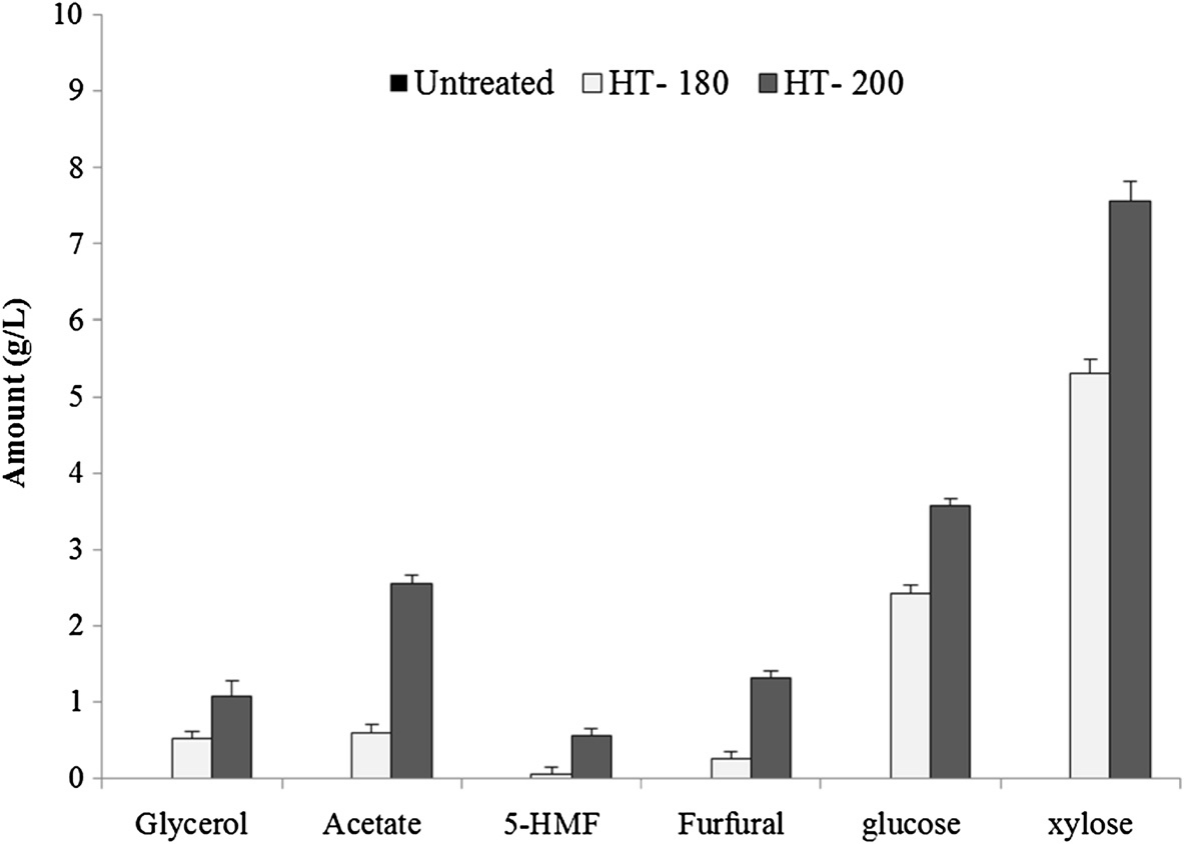
**Characterization of the liquor obtained after HT of wheat bran depicting fermentation inhibitor compounds and solubilized sugars.**

The concentration of solubilized sugars together with fermentation inhibitors is shown in Figure 5. Mass balances (shown in Figure 3A) together with details of xylan solubility by HPSEC (above) provide information of the effect of HT on carbohydrate accessibility and its saccharification. The liquors contained mostly arabinoxylan-derived xylose (3.5 and 7.6 g/L; 180°C and 200°C, respectively) and glucose (2.4 and 5.3 g/L; 180°C and 200°C, respectively; Figure 5). Small quantities of xylose-associated fragments were also detected in the liquors using NMR and increased in line with the severity of pretreatment (data not shown). This is consistent with previous reports on HT wheat straw [27,17,15] reflecting their release from the solid residues during pretreatment, followed by further degradation into monomeric xylose or converted to furfural derivatives (Figure 5). The presence of these inhibitors is undesirable because they are toxic to yeast; of greater significance is the release of acetate from acetylated xylans which exhibits a higher level of toxicity [38]. Data presented here indicate that the amounts of degradation products are expected to have a limited effect on fermentation. The most sensitive yeast strains decrease fermentation rates when mixtures of inhibitors reach 1.2 g/L 5-HMF, 0.7 g/L furfural and 6 g/L for acetic acid [39]; and most strains are only inhibited at twice this concentration. The maximum concentration of furfural at 200°C was just below this limit at 1.31 g/L therefore not expected to have any deleterious effects; however, large batch fermentations will need to be conducted in order to verify this.

### Simultaneous saccharification and fermentation (SSF) of HT wheat bran

**Figure 6:**
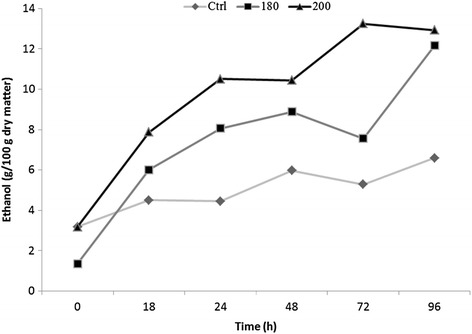
**Shake flask SSFs with Ethanol Red ® culture, a substrate loading of 10% and cellulase load corresponding to 20 FPU g/L dry cellulosic source performed at 35°C for 96 h.**

SSF of raw and pretreated wheat bran was carried out in order to confirm the beneficial effect of HT on ethanol yields. SSF was performed with CTec2® and Ethanol Red® yeast with a substrate loading of 10%, a cellulase loading of 20 FPU/g dry cellulosic source, performed at 35°C for 96 h. Figure 6 depicts the amount of ethanol in produced using SSF without added nutrients or aeration. The theoretical ethanol yield possible was 10.5, 12.5 and 13.7 g per 100 g dry bran for the control, HT at 180°C and HT at 200°C, respectively (calculated from the glucose composition in Table 2). A high ethanol conversion (of 97.5% and 96.5% of the theoretical) was obtained at both pretreatment temperatures (180°C and 200°C, respectively; Figure 6). The least amount of ethanol was produced in the untreated control bran (Figure 6) and represented 63% of the theoretical yield. Ethanol yield of 85% of theoretical has been obtained by acid hydrolysis (1% H_2_SO_4_) of wheat bran followed by enzymatic hydrolysis with Celluclast and Ultraflo, fermented using baker’s yeast (1 g/L) [23]. However, in that study, only acid-pretreated filtrates were fermented and very high concentrations of enzyme were used. In the present study, a commercial preparation of Cellic CTec2 was used which contained enzyme cocktails specifically developed for the efficient conversion of cellulose and also contained some hemicellulosic activity (www.bioenergy.novozymes.com). As such, only half the enzyme dosage was required (equivalent to 1% w/w; g enzyme/g cellulose). Similar findings were also reported for SSF of corn fibre, cobs and stover using Cellic CTec2 whereby the production of ethanol increased from 5% (v/v) to 7.2% (v/v) [22]. Fermentations were conducted using *Saccharomyces cerevisiae* (Ethanol Red®) which is already being used in distilleries for increased ethanol production. However, results from a comparative study [40] suggested that under optimum conditions the differences between ethanol yields from Ethanol Red and baker’s yeast were negligible; although the levels of acetic acid produced by Ethanol Red yeast during fermentation were significantly lower. Data presented here indicate that wheat bran is a good feedstock for conversion to ethanol considering that SSF of the untreated bran resulted in 63% of theoretical yield from glucose (cf. wheat straw) [17], probably reflecting the relatively lower level of lignification. It also suggests that the conditions used for HT were more than adequate, even at 180°C to destabilize the cell wall polymer for the efficient enzyme conversion to ethanol and relatively low concentrations of inhibitors.

## Conclusions

Hydrothermal pretreatment of destarched wheat bran released cell wall arabinoxylans, cinnamic acids and breakdown products including furfural and acetic acid. Up to 68% of the pretreated biomass could be further solubilized in alkali mostly as arabinoxylans. These exhibited significant thermochemically induced depolymerization; the level of arabinose substitution also decreased with increasing alkali concentrations. The residues contained mostly cellulosic glucose and lignin. Their simultaneous saccharification and fermentation resulted in ethanol yields of up to 97.5% theoretical. The depolymerization of the hemicelluloses would have been augmented by the considerable reduction in ferulic acid cross-links, thus contributing towards the accessibility of cellulases during enzymolysis resulting in enhanced saccharification. The reduction in cinnamic acids by selected breeding or biotechnological approaches could provide a useful basis for improved saccharification and fractionation of wheat bran polysaccharides.

## Methods

### Raw material

Milled wheat bran (WB; *Triticum aestivum* L. cv. Avalon) was obtained from G.R. Wright and Sons Ltd, Middlesex, UK and following dry matter determination stored in the freezer until use.

### Starch hydrolysis

Hydrothermal pretreatment was performed on destarched and washed wheat bran (Figure 1). Wheat bran was treated in a two-step enzymatic hydrolysis consisting of liquefaction by thermostable α-amylases (Termamyl 120 L) and saccharification by amyloglucosidase (AMG 300 L) to remove the starch. The hydrolysis was performed at a bran/water ratio of 1:5. Approximately 6 kg of bran (four batches) were hydrolysed using a Buchi Rotavapor R-153 (Büchi Labortechnik AG, Flawil, Switzerland). Firstly, bran slurry supplemented with 4 mL Termamyl 120 L/kg slurry was treated at 85°C and pH 6 for 4 h. Secondly, AMG 300 L was added at a ratio of 12 mL/kg of slurry from the first step at pH 5 and the slurry treated at 55°C for 48 h to ensure total starch digestion. The slurry was filtered through a Munktell filter paper (quality 5), and the solid material was washed with its own volume of deionized water (×3).

### Hydrothermal pretreatment (HT)

For each pretreatment, 400 g (dry weight) of destarched bran was loaded into the reactor (solid-to-water-ratio 1:10) and water was pumped through the packed material bed at a circulation flow rate of 4–5 L/min. The reactor (inner diameter 106 mm, material AISI 316) had a total volume of 6 L, with an electric heater and liquid phase circulation pump (Micropump Inc. Series 2200. The destarched bran was then pretreated at two holding temperatures (180°C/1.1 MPa and 200°C/1.55 MPa) for 10 and 15 min, respectively.

### Analysis of raw and pretreated material

#### Dry matter determination

The dry matter (DM) of the samples was determined by weighing triplicate samples in a Mettler Toledo LP16 Infrared Dryer balance (Mettler Toledo Ltd., Beaumont Leys, Leicester, UK) as described in [17].

### Preparation of alcohol insoluble residues (AIR)

AIRs were prepared from the control and pretreated wheat bran as described in [17].

### Cell wall fractionation

Sequential extraction (in progressively stronger alkali) was conducted by a modification of the method in [26]. Pretreated AIRs together with the control (2 g) were suspended in hot water (60°C, 200 mL) and cell wall components extracted (shaking, 2 h, 25°C). The extracts were centrifuged (10,000 × g, 1 h) and the supernatant filtered through Whatman GF/C filter paper (Whatman, Maidstone, UK) and freeze dried. The insoluble residue was further extracted with 0.5 mol/L KOH with 20 mmol/L NaBH_4_ for 2 h (shaking, 25°C) centrifuged (10,000 × g, 60 min) and the supernatant filtered. The filtrate was first neutralized with acetic acid, extensively dialyzed (tubing size 30/32″, Medicell International, London, UK, 7 days, changing three times daily) and then freeze dried. The residue was further extracted as above in 1 and 4 mol/L KOH containing 20 mmol/L NaBH_4_, filtered and neutralized. All the filtrates were freeze dried as above following dialysis. The freeze-dried extracts, insoluble residues and AIRs were biochemically analysed in duplicate.

### Carbohydrate analysis

Sugars were released from the fractions by hydrolysis with H_2_SO_4_ (*w* = 72%) for 3 h, followed by dilution to 1 mol/L (Saemen hydrolysis). Hydrolyzed monosaccharides were analysed as their alditol acetates by GC as described in [17].

### Starch analysis

Samples were analysed for starch using the Megazyme total starch kit (α-amylase K-TSTA 05/2008; Megazyme International Ireland Ltd, Ireland, UK) according to the manufacturer’s instructions. The analyses were performed in triplicate.

### Molecular weight (Mw) distributions

Freeze-dried alkali fractions (2 mg) were dissolved in ammonia solution (*w* = 30%) and heated (60°C, 1 h) to solubilize the extracts. HPSEC was performed on a Dionex (Sunnyvale, USA) Ultimate 3000 UPLC system equipped with three TSKgel SuperAW columns (6.0 mm ID × 150 mm per column; 4 μm) in series (SuperAW4000, SuperAW3000, SuperAW2500; Tosoh Bioscience, Stuttgart, Germany), in combination with a guard column (Tosoh Bioscience, Stuttgart, Germany). Columns were eluted at 40°C with 0.2 mol/L sodium nitrate at 0.6 mL/min. The eluate was monitored with a Shodex RI-101 (Kawasaki, Japan) refractive index (RI) detector. Dextran standards (Sigma-Aldrich, Poole, Dorset, UK) were used for Mw calibration.

### Lignin and phenolic acids

Klason lignin was quantified gravimetrically as described in [17]. The samples were analysed in triplicate. The total alkali-extractable hydroxycinnamate content of the AIRs and insoluble residues after fractionation was determined by saponification of 5 mg of sample in 4 mol/L sodium hydroxide as described in [17].

### Acetic acid, furfural and hydroxymethylfurfural (HMF)

Degradation products were quantified in the pretreated liquids using a Shimadzu HPLC system (Hertogenbosch, The Netherlands) with column oven CTO-10A-vp and Autoinjector SIL-10 AD-vp equipped with a guard column (Bio-Rad H cartridge, Bio-Rad Laboratories Ltd, Hemel Hempstead, Herts., UK) and an Aminex HPX-87H column (300 × 7.8 mm; Bio-Rad Laboratories Ltd, Hemel Hempstead, Herts., UK). Elution took place at 80°C with 5 mmol/L H_2_SO_4_ at 0.6 mL/min. The eluate was monitored using a Refractive Index detector RID-10A (Shimadzu, Kyoto, Japan).

### Light and autofluorescence microscopy

AIRs of samples were observed in their native state and photographed using a Wild M8 stereomicroscope. For higher magnification, samples were observed with an Olympus BX60 microscope (Olympus, Tokyo, Japan) interfaced with a ProRes© Capture Pro 2.1 camera and software (Jenoptik, Jena, Germany). The autofluorescence was recorded using the UV filter cube with a filter cube configuration of excitation filter band pass 330–385 nm, barrier filter 420 nm.

### Simultaneous saccharification and fermentation (SSF)

#### Yeast cultivation

Ethanol Red® was prepared after pre-culturing aerobically at 30°C in yeast propagating medium (YP; Difco, Lawrence, Kansas, USA) containing yeast extract 1%, peptone 2% and glucose 2%. The yeast cells were transferred to YP medium supplemented with 5% glucose and cultured aerobically at 30°C overnight. The cells were harvested, washed with 0.9% NaCl solution to remove any residual nutrients, resuspended in ten times concentrated yeast nitrogen base (YNB; Formedium, Hunstanton, UK) which contained no glucose and inoculated (10% v/v) in fermentation flasks to give initial yeast concentration of 5 g/L (dry weight). Active cultures for inoculation were prepared by growing the organism in YM (growth medium Sigma-Aldrich, Poole, Dorset, UK) supplemented with: 0.3% yeast extract, 0.3% malt extract, 0.5% peptone and 1% dextrose (Difco, Lawrence, Kansas, USA) shaking (rotary shaker, 200 rpm, 18 h, 25°C).

### Fermentation

Fermentations were performed on washed pretreated wheat bran slurry (10%) in closed universal bottles (20 mL, VWR International Ltd, Leicester, Leicestershire, UK) containing 9 mL of YNB without nutrients in sodium acetate buffer pH 5.0 (autoclaved, 121°C, 90 min), 1 mL of YNB in sodium acetate buffer pH 5.0 (autoclaved, 121°C/90 min) and 1 mL of yeast cell suspension (10%). A cellulase load (Cellic® CTec2, Novozymes, Bagsvaerd, Denmark) corresponding to 20 FPU/g dry cellulosic source was transferred to the system prior to the SSF process which was performed at 35°C for 96 h (anaerobically, shaking). After the reaction time, enzyme deactivation (100°C, 10 min) was followed by quantification using HPLC (Waters®, Waters Ltd, Hertfordshire, UK) equipped with a refractive index detector using a Bio-Rad Aminex HPX-87P column (Bio-Rad Laboratories Ltd, Hemel Hempstead, UK) at 65°C and MQ water (Millipore®, Millipore Ltd, Watford, UK) as mobile phase at a flow rate of 0.6 mL/min. Positive (copier paper) and negative (MQ water) controls were prepared using exactly the same procedure as the test samples, and the ethanol peaks in the negative controls were subtracted from the test peaks prior to tabulation. Ethanol values were expressed as theoretical yield (from carbohydrate data in Table 2).

### Calculations

Calculations were performed using Microsoft Office Excel (2010; Microsoft, Berkshire, UK). The percentage carbohydrate yields were calculated from the monomeric amounts given in Table 1. Percentage changes as a result of the pretreatment have been calculated based on the original starting material (control AIR). The closing mass balance was generated from the dry biomass entering the hydrothermal pretreatment (HT) system, the amount of water added and the final mass of the solid residue obtained following pretreatment together with the liquid fractions. The key sugars were calculated as monomeric equivalents present in the control and pretreated AIRs, taking into account the dry matter (DM) content and back calculated to the original starting material entering the pretreatment stream. As a starting point, the amount given in Table 1 was multiplied by the DM content to determine the starting amount of each sugar. So for each pretreatment, key sugars were calculated as follows:

For example, for glucose yield

$$ \left(g/ kg\right)=\frac{\mathrm{HT}\ \mathrm{biomass}\ (g)\ast 1,000\ast \mathrm{D}\mathrm{M}\ast \mathrm{amount}\ \mathrm{of}\ \mathrm{glucose}\ \mathrm{in}\ \mathrm{H}\mathrm{T}\ \mathrm{AIR}\ (g)}{\mathrm{Starting}\ \mathrm{biomass}\ (g)} $$

The ethanol yields were calculated as follows:

Ethanol (*g*) = Glucose present (%) × Weight of material × 0.511 (factor for ethanol conversion) × 1.111 (water of hydrolysis).

## Abbreviations

AIR: alcohol insoluble residue
DiFA: diferulic acid
DM: dry matter
HT: hydrothermal pretreatment
HPSEC: high-performance size exclusion chromatography
HW: hot water extraction
Mw: molecular weight
SSF: simultaneous saccharification and fermentation
